# Preoperative fistula diagnostics in male anorectal malformations after colostomy: a single-center experience

**DOI:** 10.1186/s12880-023-01105-3

**Published:** 2023-09-25

**Authors:** Jianxi Bai, Bing Zhang, Kaiwu Lin

**Affiliations:** 1 Pediatric Surgery Department, Fujian Children’s Hospital, Fuzhou City, Fujian Province China; 2 Pediatric Surgery Department, Fujian Branch of Shanghai Children’s Medical Center, Fuzhou City, Fujian Province China; 3 Pediatric Surgery Department, Fujian Maternity and Child Health Hospital, Affiliated Hospital of Fujian Medical University, Fuzhou City, Fujian Province China; 4 Radiology Department, Fujian Provincial Maternity and Children Hospital, 18 Daoshan Road, Gulou District, Fuzhou City, Fujian Province China

**Keywords:** MRI, Colostogram, Anorectal malformations, Fistula, VCUG

## Abstract

**Background:**

Accurate preoperative fistula diagnostics in male anorectal malformations (ARM) after colostomy are of great significance. We reviewed our institutional experiences and explored methods for improving the preoperative diagnostic accuracy of fistulas in males with ARMs after colostomy.

**Methods:**

A retrospective analysis was performed on males with ARMs after colostomy admitted to our hospital from January 2015 to June 2022. All patients underwent magnetic resonance imaging (MRI) and high-pressure colostogram (HPC) before anorectal reconstruction. Patients with no fistula as diagnosed by both modalities underwent a voiding cystourethrogram (VCUG). General information, imaging results and surgical results were recorded.

**Results:**

Sixty-nine males with ARMs after colostomy were included. Age at the time of examination was 52 ~ 213 days, and the median age was 89 days. The Krickenbeck classification according to surgical results included rectovesical fistula (n = 19), rectoprostatic fistula (n = 24), rectobulbar fistula (n = 19) and no fistula (n = 7). There was no significant difference in the diagnostic accuracy between MRI and HPC for different types of ARMs. For determining the location of the fistula, compared to surgery, HPC (76.8%, 53/69) performed significantly better than MRI (60.9%, 42/69) (p = 0.043). Sixteen patients diagnosed as having no fistula by MRI or HPC underwent a VCUG, and in 14 patients, the results were comfirmed. However, there were 2 cases of rectoprostatic fistula that were not correctly diagnosed.

**Conclusion:**

High-pressure colostogram has greater accuracy than MRI in the diagnosis of fistula type in males with ARMs after colostomy. For patients diagnosed with no fistula by both methods, VCUG reduces the risk of false-negative exclusion, and rectoprostatic fistula should be considered during the operation.

## Introduction

Anorectal malformations (ARMs) are common congenital malformations in newborns, with an incidence rate of approximately 1:5000 [1]. ARMs cover a broad spectrum of diseases, including anal stenosis, ventral anus, anal atresia (with and without fistula) and the full spectrum of cloacal malformations. It is often accompanied by abnormalities of the cardiovascular, gastrointestinal, spinal and genitourinary systems [2]. For children without obvious fistula in the perineum, colostomy is often required first [3]. Assessment of the presence or absence of any fistula and the type and location of the fistula, if present, before surgery is of great significance for the choice of surgery [4], postoperative efficacy and prevention of complications [5–7].

Magnetic resonance imaging (MRI) and fluoroscopy are the two mainassessment methods for ARMs before definitive surgery [8]. MRI is nonradioactive and noninvasive, and has great advantages in displaying soft tissues. It has been recognized that MRI can clearly reveal anomalies associated with ARMs compared with other modalities [9]. MRI is advocated as a promising “one-stop shop” modality [8]. High-pressure colostogram (HPC) is considered the most effective method for diagnosing fistulas, but there is a lack of large-scale comparative study and the previous studies have some methodological issues [10]. Voiding cystourethrogram (VCUG) is helpful in the diagnosis of fistula [7], but there is controversy concerning when it should be performed [8]. Performing HPC and VCUG on all patients increases the dose of radiation and is unnecessary. We performed VCUG for children diagnosed with no fistula by both MRI or HPC and improved preoperative diagnostic accuracy of fistulas in males with ARMs after colostomy.

## Methods

### Patients

The Ethics Committee of Fujian Provincial Maternity and Children Hospital approved this retrospective single-center study with and waivd informed consent. A retrospective analysis was performed on the males with ARMs after colostomy admitted to our hospital from January 2015 to June 2022. All children underwent MRI and HPC before anorectal reconstruction, and ARMs were confirmed by surgery according to the diagnostic criteria of the Krickenbeck classification. MRI and HPC were completed within 2 days, and MRI was performed before HPC. Patients with no fistula diagnosed by both MRI and HPC underwent VCUG.

### MRI

MRI was conducted using a 1.5 T unit (General Electrics, Signa, HDe). Abdominal and pelvic scans were performed with body coils. The imaging included a T2-weighted (T2W) fast recovery fast spin echo [FRFSE, slice thickness = 3 mm, repetition time (TR) 3500 ~ 5000 ms, echo time (TE) 95 ~ 110 ms] and T1-weighted (T1W) spin echo [SE, slice thickness = 3 mm, TR 500 ~ 600 ms, TE 25 ms] sequence in three directions. Uncooperative children were given 10% chloral hydrate (0.5 mL/kg body weight, po.) prior to the scan. MRI distal colostograms were not performed.

### High-pressure colostogram

High-pressure colostograms were conducted using a digital gastrointestinal machine (General Electrics, Precision, RXi). A Foley catheter was inserted into the distal rectal pouch, and the balloon was filled with normal saline and pulled back to occlude the stoma. The distal colorectal pouch was treated with an enema with iopromide to maintain a certain pressure and obtain optimal distension, resulting in imaging of a possible fistula.

### VCUG

VCUG was conducted using a digital gastrointestinal machine (General Electrics, Precision, RXi). A 6-or 8-Fr feeding tube was inserted from the external urethral orifice into the bladder. The bladder was filled with diluted iopromide. Under fluoroscopy (real-time radiographs), the contrast agent was observed entering the bladder and during voiding. The presence or absence and the location of the fistula were evaluated.

### Evaluation of fistula

Images were independently reviewed by two pediatric radiologists with more than 10 years of experience who were unaware of the intraoperative findings. Information regarding the anal opening, which was clinically visible, was provided to both readers. Readers were asked to evaluat the presence and location of the fistula. For MRI, differentiation between normal colon and fistula was based on the layered aspect of the bowel segment. If the different layers (mucosa, submucosa and muscularis) were discernable, it was classified as a normal bowel that could be used for anastomosis. If there were no layers visible, it was classified as a fistula [11]. The classification criteria on the images of high-pressure colostograms were taken from the literature [12]. When opinions were divided, the readers discussed and reached an agreement.

### Statistical analysis

McNemar’s test was used to compare the diagnostic performance of MRI vs. high-pressure colostogram for the detection of fistula in different types of ARMs. The Chi-square test was used to compare the proportion of correct diagnoses of the presence and location of fistula (against the reference standard described above) made with MRI or conventional fluoroscopic studies. P values of less than 0.05 were considered significant. All statistical analyses were performed using SPSS 16.0.

## Results

### Patients

Sixty-nine males with ARMs after colostomy were included. The age at the time of examination was 52 ~ 213 days, and the median age was 89 days. The Krickenbeck classification according to surgical results included rectovesical fistula (n = 19), rectoprostatic fistula (n = 24), rectobulbar fistula (n = 19) and no fistula (n = 7).

### Diagnostic performance for detecting fistulas

**Fig. 1:**
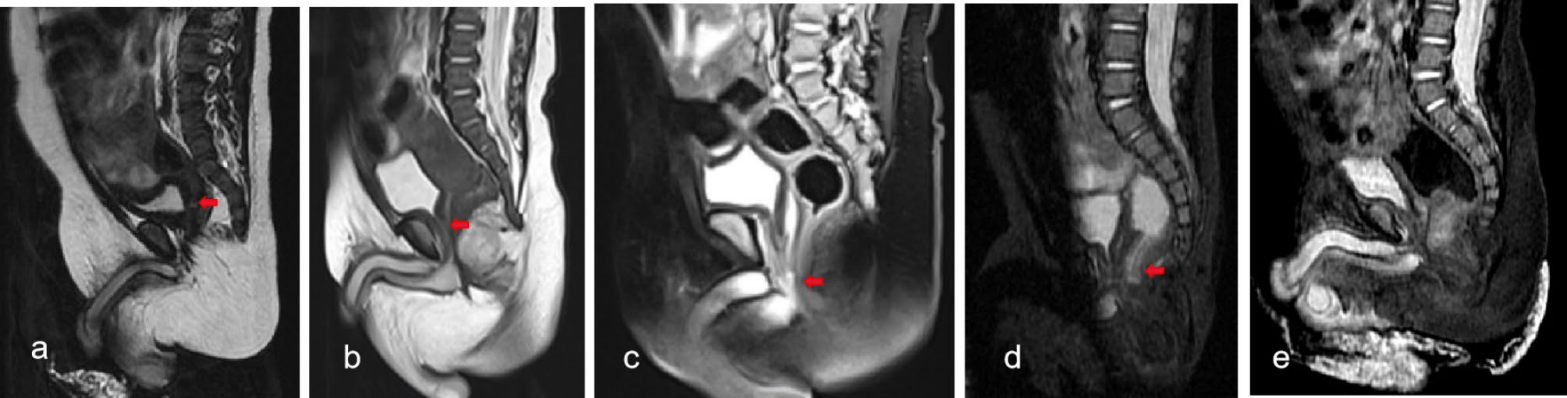
Fistula (red arrow) in MRI. **(a)** Rectovesical fistula. **(b)** Rectoprostatic fistula. **(c)** Rectobulbar fistula. **(d)** Fistula incompletely revealed. e.No fistula

For MRI (Figs. 1), 35 cases of fistula were clearly detected (Fig. 1a-c). Seven cases of invisible fistulas were conformed to have no fistula by operation (Fig. 1d); 10 cases of fistula were incompletely revealed (Fig. 1e), with the image showing the proximal or distal sides of the tract and continuous interruption of fistula signal, so the location of fistula could not be diagnosed. The remaining 17 cases of invisible fistula were misdiagnosed. There was no significant difference in the diagnostic accuracy of MRI for different types of fistulas.

**Fig. 2:**
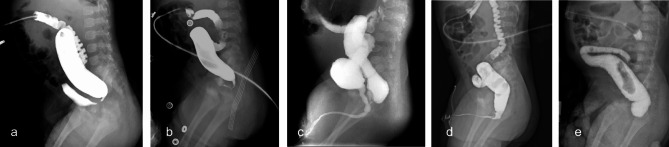
Fistula in high-pressure colostogram. **(a)** Rectovesical fistula. **(b)** Rectoprostatic fistula. **(c)** Rectobulbar fistula with urethra and bladder revealed. **(d)** Rectobulbar fistula with urethra revealed. e.No fistula

For the high-pressure colostogram (Figs. 2), 46 cases of fistula were clearly detected. Among them, in 14 cases of rectovesical fistula, the contrast agent directly entered the bladder through the fistula (Fig. 2a); 15 cases of rectoprostatic fistula showed posterior urethra and countercurrent flow into the bladder, and the anterior urethra began to develop after continuous injection (Fig. 2b); in 16 cases of rectobulbar fistula, 13 cases had a fistula opening at the curvature of the urethra with anterior and posterior urethra and bladder development (Fig. 2c), and the other 3 cases had a fistula opening at the distal end of the urethra with only urethra development (Fig. 2d). Seven cases of invisible fistula were proven to have no fistula by operation (Fig. 2e); the remaining 16 cases of invisible fistula were misdiagnosed. There was no significant difference in the diagnostic accuracy for different types of fistulas.

For determining the presence of fistula, high-pressure colostogram (76.8%, 53/69) and MRI (75.3%, 52/69) brought identical results (p = 0.692). However, for determining the location of the fistula (Table 1), compared to surgery, high-pressure colostogram (76.8%, 53/69) performed significantly better than MRI (60.9%, 42/69) (p = 0.043). For each different type of fistula, there was no significant difference in the diagnostic accuracy by MRI vs. high-pressure colostogram (Tables 2, 3 and 4).

**Table 1:** Total number of correct diagnoses by MRI or high-pressure colostogram

Krickenbeck classification	MRI	high-pressure colostogram
Rectovesical fistula (n = 19)	11	15
Bulbar recto-urethral fistula (n = 19)	12	15
Prostatic recto-urethral fistula (n = 24)	12	16
no fistula (n = 7)	7	7
Total (n = 69)	42	53

**Table 2:** Total number of correct diagnoses for rectovesical fistula by MRI vs. high-pressure colostogram

MRI	high-pressure colostogram		Total
	True	False	
True	8	3	11
False	7	1	8
Total	15	4	19

χ^2^=0.9, p = 0.343.

**Table 3:** Total number of correct diagnoses for rectoprostatic fistula by MRI vs. high-pressure colostogram

MRI	high-pressure colostogram		Total
	True	False	
True	10	2	12
False	5	7	12
Total	15	9	24

χ^2^=0.571, p = 0.450.

**Table 4:** Total number of correct diagnoses for rectobulbar fistula by MRI vs. high-pressure colostogram

MRI	high-pressure colostogram		Total
	True	False	
True	10	2	12
False	6	1	7
Total	16	3	19

χ^2^=1.125, p = 0.289.

**Fig. 3:**
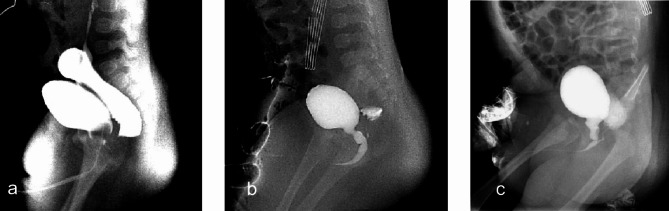
Fistula diagnosed by VCUG. **(a)** Rectovesical fistula. **(b)** Rectoprostatic fistula. **(c)** Rectobulbar fistula

Sixteen cases diagnosed as no fistula by both MRI and high-pressure colostogram underwent VCUG (Fig. 3). By VCUG, 7 cases of no fistula, 1 case of rectovesical fistula (Fig. 3a), 5 cases of rectoprostatic fistula (Fig. 3b) and 1 case of rectobulbar fistula (Fig. 3c) were correctly diagnosed; 2 cases of rectoprostatic fistula were still diagnosed as no fistula and thus missed. Among 9 cases with double-negative MRI and HPC results, rectoprostatic fistula had the highest proportion among the three types of urinary fistula (1/19, 7/24, 1/19; p = 0.034).

## Discussion

This study reviewed a large sample size of males with ARMs after colostomy, compared the performance of MRI and high-pressure colostogram, and found that the accuracy of MRI in diagnosing fistula was not as high as previously reported in the literature and that high-pressure colostogram was superior to MRI in the accurate diagnosis of fistula in males with ARMs after colostomy. Both modalities had a relatively high rate of missed diagnosis of rectoprostatic fistula, and the addition of VCUG helped to improve the accuracy of fistula diagnosis. MRI and fluoroscopy are the main imaging methods for diagnosing ARMs with fistula [9, 13, 14]. Compared with other examination methods, MRI is considered a promising one-stop examination method [8]. It has been recognized that MRI has advantages in revealing anomalies associated with ARMs, such as presacral masses; spinal, sacral and vertebral anomalies; and genitourinary malformations. Some previous studies have reported the diagnostic performance of MRI in fistula evaluation compared with other imaging modalities [7, 11, 15–17]. However, in these comparative studies, colonography, voiding cystourethrogram, and fistulography were compared with MRI as one modality and were not differentiated. Howerer, the scope of the application of these examination methods is different. Moreover, children with visible fistula, such as perineal fistula and vestibular fistula, should be excluded since these types of fistulas do not need any imaging modality to determine the location, because it can be seen on physical exam [10]. However for rectovesical fistula, rectourethral fistula, rectovaginal fistula, etc., there is a greater possibility of urethral injuries when making a circumferential incision around the fistula during the operation. Therefore, it is of great significance to correctly evaluate fistulas in males with ARMs after colostomy before performing definitive surgery.

MRI is mainly used to visualize the fistula with sagittal and axial FRFSE/FSET2WI sequences, and sagittal is the best view, showing linear or tubular shadows between the lower end of the rectum and the perineum, urethra, bladder or vagina with high or slightly high T2WI signals. Our results showed that the diagnostic accuracy of MRI for ARM fistulas was lower than that of high-pressure colostogram (76.8% vs. 60.9%; P = 0.043). However, our conclusion is slightly different from that of other studies. Maarten G. Thomeer et al. [11] compared MRI and colostography/fistulography in neonates with ARMs. Their results showed that MRI and imaging/tomography predicted fistulas in 88% (29/33) and 61% (20/33) of cases, respectively (p = 0.012). Yang Zhan et al. [17] reported that the fistula type was correctly identified by MRI and colostography/fistulography in 91.7% (22/24) and 62.5% (15/24), respectively (p = 0.039). This may be related to the following factors: (1) Their research did not exclude children with visible fistula. The detection accuracy of either of the two methods (MRI or fistulography) for those fistulas is almost 100%. (2) The examination time may affect the diagnosis. In the early postnatal period, the rectal blind end is a large expansion filled with meconium, and the fistula contains more lipid components. MRI easily to displays the location and course of the fistula. However, after colostomy, the fistula contents are reduced, the fistula is thinner, the location is hidden, and the scanning layer is too thick, which leads to a fail or unclear display of the fistula or a poor display of the fistula when it was blocked, which affects the accuracy of comparison between MRI and other methods. (3) The studies had a lack of unified MRI protocol and technology. Another limitation of MRI is the current limited imaging resolution. We observed that in 10 cases, the fistula was incompletely revealed, and the location of the fistula entry was not clearly displayed, leading to a reduced efficiency of MRI in judging fistula locations. To improve the display of fistulas, MRI distal colostogram has been studied. Lucie Kavalcova et al. [14] inserted a thin precise tube through the fistula and filled the fistula with a line (10 ml/kg) for the MRI-FG. However, modified MRI had identical results as contrast studies in visualizing the fistula and rectum length and course of 25 patients. More recent studies have abandoned the modified methods and advocated standard eating and sleeping (“eating and wrapping”) pelvic MRI, which does not involve expanding the distal colon ring or injecting the perineal fistula [8]. In addition, whether more advanced MRI instruments and technologies, such as high-Tesla MRI, can help improve the accuracy of fistula diagnosis needs further research.

For high-pressure colostograms, there were 16 false-negative cases, and the proportion (16/62) was not low. It has been reported that the diagnoses missed by colostogram may be related to the inability to open the fistula caused by insufficient pressure [18]. The importance of the correct operation of high-pressure colostograms has been emphasized repeatedly [5, 6, 18–21]. Recently, the European Society of Pediatric Radiology (ERSP) emphasized seven technical points of high-pressure colostograms. One is that distention of the whole distal loop to reduce the risk of false-negative exclusion of a fistula [8]. Referring to these points, we think it is relatively difficult to evaluate whether the pressure is sufficient. In our study, pressure control depended on indirect signs such as optimal distension of the rectal pouch, which involved a certain degree of subjectivity. In addition, increasing the pressure may increase the possibility of reopening the blocked fistula after colostomy, and it also increases the risk of intestinal perforation [12, 20]. The reported incidence of bowel perforation is approximately 2% [12, 22]. Although no cases occurred in our study, the risk of bowel perforation caused by high-pressure colostogram should still be considered.

Initial VCUG (before any surgery) is considered to be as accurate as distal colostogram in the evaluation of male patients with ARMs [23]. However, in males with ostomy, the accuracy of VCUG in diagnosing fistulas was shown to be not statistically superior to that of high-pressure colostogram [7]. However, it is recommended that VCUG be used as a supplementary examination, depending on the underlying condition [6, 21]. We added VCUG for cases diagnosed as having no fistula by both MRI and colostogram, which improved the accuracy of fistula diagnosis. Therefore, we suggest that for children diagnosed with no fistula by MRI or HPC, VCUG should be used to reduce the risk of a false-negative exclusion of a fistula.

We also observed that in the case of false negative by both MRI and high-pressure colostogram, the rate of rectal prostate fistula was the highest, and evenwhen VCUG was added, there were still 2 missed diagnoses. This may be related to the thinness of the fistula which is difficult to find and open. Therefore, for children diagnosed with no fistula, rectoprostatic fistula should still be considered during the operation.

Our research has some limitations. This study is a retrospective analysis, and the specific conditions of the inspection process are difficult to trace. MRI techniques evolved significantly during the study period, which may underestimate the value of MRI in early patients. High-pressure colostogramis is a dynamic study, and the presence of a tiny fistula may not be captured in the images.

## Conclusion

MRI is not superior to high-pressure colostogram in the accurate diagnosis of fistula type in males with ARMs after colostomy. For children diagnosed with no fistula by both methods, VCUG is a good supplement, and rectoprostatic fistula should be considered during the operation.

## Data Availability

The datasets used and analysed during the current study are available from the corresponding author on reasonable request.

## Abbreviations

ARMs: Anorectal malformations
HPC: High-pressure colostogram
MRI: Magnetic resonance imaging
VCUG: Voiding cystourethrogram
